# Thickness-modulated tungsten–carbon superconducting nanostructures grown by focused ion beam induced deposition for vortex pinning up to high magnetic fields

**DOI:** 10.3762/bjnano.7.162

**Published:** 2016-11-14

**Authors:** Ismael García Serrano, Javier Sesé, Isabel Guillamón, Hermann Suderow, Sebastián Vieira, Manuel Ricardo Ibarra, José María De Teresa

**Affiliations:** 1 Laboratorio de Microscopías Avanzadas (LMA), Instituto de Nanociencia de Aragón (INA), Universidad de Zaragoza, 50018 Zaragoza, Spain; 2 Departamento de Física de la Materia Condensada, Universidad de Zaragoza, 50009 Zaragoza, Spain; 3 Laboratorio de Bajas Temperaturas, Unidad Asociada UAM/CSIC, Instituto Nicolás Cabrera, Condensed Matter Physics Center (IFIMAC), Departa-mento de Física de la Materia Condensada, Universidad Autónoma de Madrid, Spain; 4 Instituto de Ciencia de Materiales de Aragón (ICMA), CSIC - Universidad de Zaragoza, 50009 Zaragoza, Spain

**Keywords:** focused ion beam induced deposition, magnetotransport, superconductivity, vortex lattice

## Abstract

We report efficient vortex pinning in thickness-modulated tungsten–carbon-based (W–C) nanostructures grown by focused ion beam induced deposition (FIBID). By using FIBID, W–C superconducting films have been created with thickness modulation properties exhibiting periodicity from 60 to 140 nm, leading to a strong pinning potential for the vortex lattice. This produces local minima in the resistivity up to high magnetic fields (2.2 T) in a broad temperature range due to commensurability effects between the pinning potential and the vortex lattice. The results show that the combination of single-step FIBID fabrication of superconducting nanostructures with built-in artificial pinning landscapes and the small intrinsic random pinning potential of this material produces strong periodic pinning potentials, maximizing the opportunities for the investigation of fundamental aspects in vortex science under changing external stimuli (e.g., temperature, magnetic field, electrical current).

## Introduction

In focused electron/ion beam induced deposition (FEBID/FIBID), a precursor molecule is dissociated by a focused electron/ion beam, producing the local growth of a deposit in a single step and with the shape determined by the electron/ion beam scan [1–4]. Materials grown by FEBID/FIBID can show a wide variety of functionalities: conductive [5], insulating [6], magnetic [7], optical [8], superconductive [9], etc. In particular, FIBID has been used to grow superconducting W–C nanodeposits with a relatively high critical temperature, *T*_c_, up to 6 K using a W(CO)_6_ precursor [9–19]. Interestingly, these W–C films are amorphous or nanocrystalline and the intrinsic pinning is low. Even small surface corrugations of just a few percent of the total thickness allow the observation of vortex-lattice matching effects by means of scanning tunneling microscopy (STM) [20–21]. Recently, De Teresa and Córdoba proposed a strategy to grow W–C films by FIBID with controlled thickness modulation [22], which opens the route for the design of specific experiments probing the behavior of the vortex lattice as a function of magnetic field, temperature and electrical current. In the present work, we exploit such a strategy to create linear-shape vortex-pinning landscapes in W–C films grown by FIBID. In sharp contrast with the use of other growth and lithography techniques that require several steps (with the risk of increasing the random pinning produced by defects), our approach permits a superconducting nanostructure to be obtained in a single step with the designed pinning landscape through the thickness modulation. This gives rise to a clean model system for the investigation of the influence of artificial vortex pinning landscapes in the superconducting properties, as shown here.

Before describing the designed vortex pinning landscape in the W–C nanostructures, let us mention some specific aspects of vortex pinning in superconductors and the influence on magnetotransport properties, which will be useful to understand our data. The application of external magnetic fields on type-II superconductors gives rise to a vortex lattice that is hexagonal in most cases [23]. In the presence of a current, vortices move under the action of the Lorentz force [24], producing dissipation and limiting its electrical current and magnetic field working ranges. Vortex motion can be hindered by pinning barriers that act on vortex motion below the thermal depinning temperature, which depends on the arrangement and size of barriers.

As a consequence, one of the mainstreams of research in superconductivity is to pin vortices and impede or reduce their motion [25–45]. In order to design vortex-pinning landscapes, electron beam lithography is commonly used to fabricate arrays of holes [33,40] or arrays of magnetic dots/lines [30,34,36,39,41], whereas selective ion implantation [29,38,41] and insertion of structural defects [35,37,43] are other common pinning strategies. The use of a focused ion beam (FIB) for enhanced vortex pinning through local removal of the superconducting material has been explored as well [40,42,46–48]. In contrast to our approach followed here, such previous work has focused on the use of FIB for milling instead of deposition and the pinning has not been observed up to high magnetic fields, as it was for our case.

Additionally, thickness modulation is known to produce vortex-pinning effects due to the dependence of the vortex energy with its length, which favors the location of vortices in the thinnest parts of the superconductor for vortices perpendicular to the film [31]. In the past, some experiments were performed to generate microscale thickness modulation by pressing diffraction gratings on superconducting foils [25] or by photolithography processes [27], which led to the observation of vortex-pinning effects at low magnetic fields (<0.02 T). Here, we present and discuss results obtained in linear pinning potentials engineered at nanoscale dimensions to observe matching effects with the vortex lattice over a broad magnetic-field range (up to 2.2 T). We underline that given the clean (low random pinning) sample growth process that already includes the artificial periodic pinning potential, the experiments described here can be hardly achieved following other strategies, explaining why the matching effects between the pinning periodicity and the vortex lattice have been observed up to high magnetic fields and in a broad temperature range.

**Figure 1:**
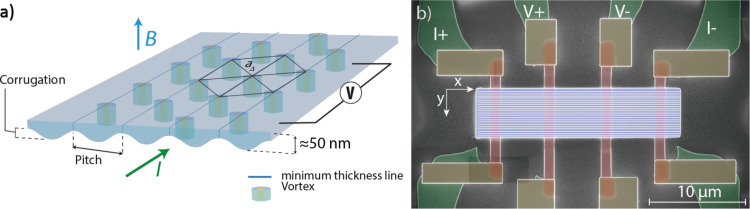
(a) Scheme of the experiment performed to measure the electrical resistance under perpendicular magnetic field. Due to the Lorentz force, the vortex lattice tends to move transversally to the low-thickness zones, which act as vortex pinning lines. (b) SEM false-colored micrograph showing the Ti pads (green), the buried contacts of Pt (red), the Pt connection to the Ti pads (brown) and the W–C deposit (blue) with the pinning grooves along the *x* direction.

Figure 1 displays the relative arrangement of the current, magnetic field and linear nanostructures in the magnetotransport experiments performed, together with a top-down scanning electron microscope (SEM) micrograph of one of the samples after growth. Given that under a fixed magnetic field applied perpendicularly to the superconducting film, vortices form perpendicular to the film surface, the application of an electrical current parallel to the grooves produces a Lorentz force perpendicular to the grooves. Consequently, the pinning potential caused by the thickness modulation (grooves) has to be overcome in order to move the vortex lattice. The intervortex distance is known to be dependent only on the applied magnetic field:

[1]
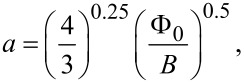


where *a* is the intervortex distance, 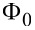
is the quantum of magnetic flux (2.07 × 10^−15^ Wb) and *B* is the magnetic flux density [24]. As an example, the application of a 1 T magnetic field implies an intervortex distance of around 50 nm. In the magnetotransport experiments, we expect to find features related to the matching between the pinning periodicity and the vortex lattice dimensions.

## Results and Discussion

### Sample growth and characterization

**Figure 2:**
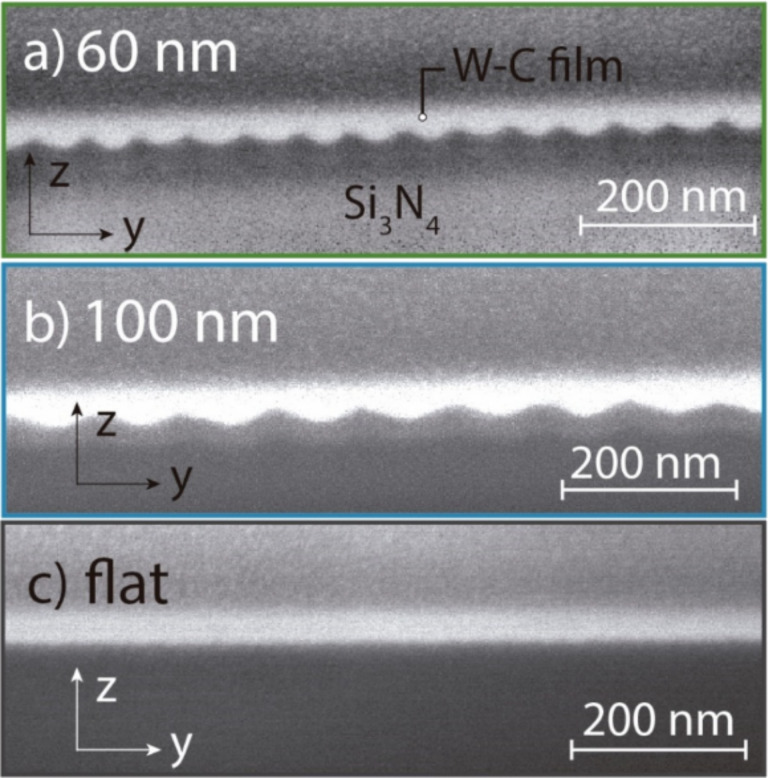
Cross-sectional SEM micrographs of a sample with 60 nm pitch (a), a sample with 100 nm pitch (b) and the flat sample (c). The *y* and *z* axes correspond to the short sides of the film and the thickness direction, respectively.

As shown in Figure 2, FIBID allows the growth of W–C films that are either flat or display engineered thickness modulation (corrugation). In the present work, the thickness corrugation arises mainly at the interface between the film and the substrate because during growth, the ion beam removes substrate material from the scanned areas [22]. During growth, the ion beam is scanned following linear paths, which produces characteristic grooves and crest–valley-structured films. The beam scan periodicity (pitch) is varied in the present work from 60 to 140 nm, which allows us to determine the effect of its commensurability with the intervortex distance.

The W–C deposits have been grown by FIBID inside commercial Helios 650 dual-beam equipment from FEI, which includes a Ga^+^ FIB column. The equipment includes a gas-injection system for the W(CO)_6_ precursor. The precursor gas is spread locally onto the substrate, where it becomes dissociated by the FIB. The composition of flat deposits has been previously studied with X-ray photoelectron spectroscopy [13], giving as a result (in atom %), W (40%), C (43%), Ga (10%) and O (7%). The nature of the deposits is amorphous, as previous STM and transmission electron microscopy studies have demonstrated [11,13]. The W–C samples have been grown on Si_3_N_4_ substrates prepatterned with Ti pads by e-beam evaporation and lift-off techniques for magnetoresistance measurements using a four-point configuration (see Figure 1b). Samples with five different periodicity values of the thickness modulation (60, 80, 100, 120 and 140 nm) and one additional flat sample without modulation for control experiments were grown. The samples have a sectional area of 0.21 ± 0.03 μm^2^, with a critical temperature of *T*_c_ = 4.40 ± 0.15 K and a room temperature resistivity of ρ_300K_ = 213 ± 47 μΩ cm.

The flat sample has been made using a 39 nm *y*-pitch, which is as large as the beam spot size. The SEM images in Figure 2 indicate the achievement of the targeted thickness modulation. In Table 1, the thickness and corrugation of the films are reported, indicating that the corrugation (difference between the maximum and minimum thickness for a given sample) increases with the pitch value. For the samples with the smaller pitches (60 and 80 nm), the corrugation is about 1/3 of the maximum thickness, whereas for the samples with higher pitches (120 and 140 nm), the corrugation is greater than 50%.

**Table 1:** Maximum and minimum thickness of the studied samples obtained from measurements in cross-sectional SEM micrographs. The corrugation is calculated as the difference between the maximum and minimum thickness. The relative corrugation (%) is calculated by dividing the corrugation into the maximum thickness and multiplying by 100. The error bars take into account the error in the measurements and differences amongst all the samples with a given pitch.

Pitch (nm)	Maximum thickness (nm)	Minimum thickness (nm)	Corrugation (nm)	Corrugation (%)

flat	48 ± 2	48 ± 2	0 ± 4	0
60	46 ± 3	31 ± 5	15 ± 8	34
80	55 ± 4	36 ± 3	19 ± 7	34
100	57 ± 5	36 ± 2	21 ± 7	37
120	60 ± 3	23 ± 3	37 ± 6	61
140	66 ± 3	31 ± 3	35 ± 6	52

**Figure 3:**
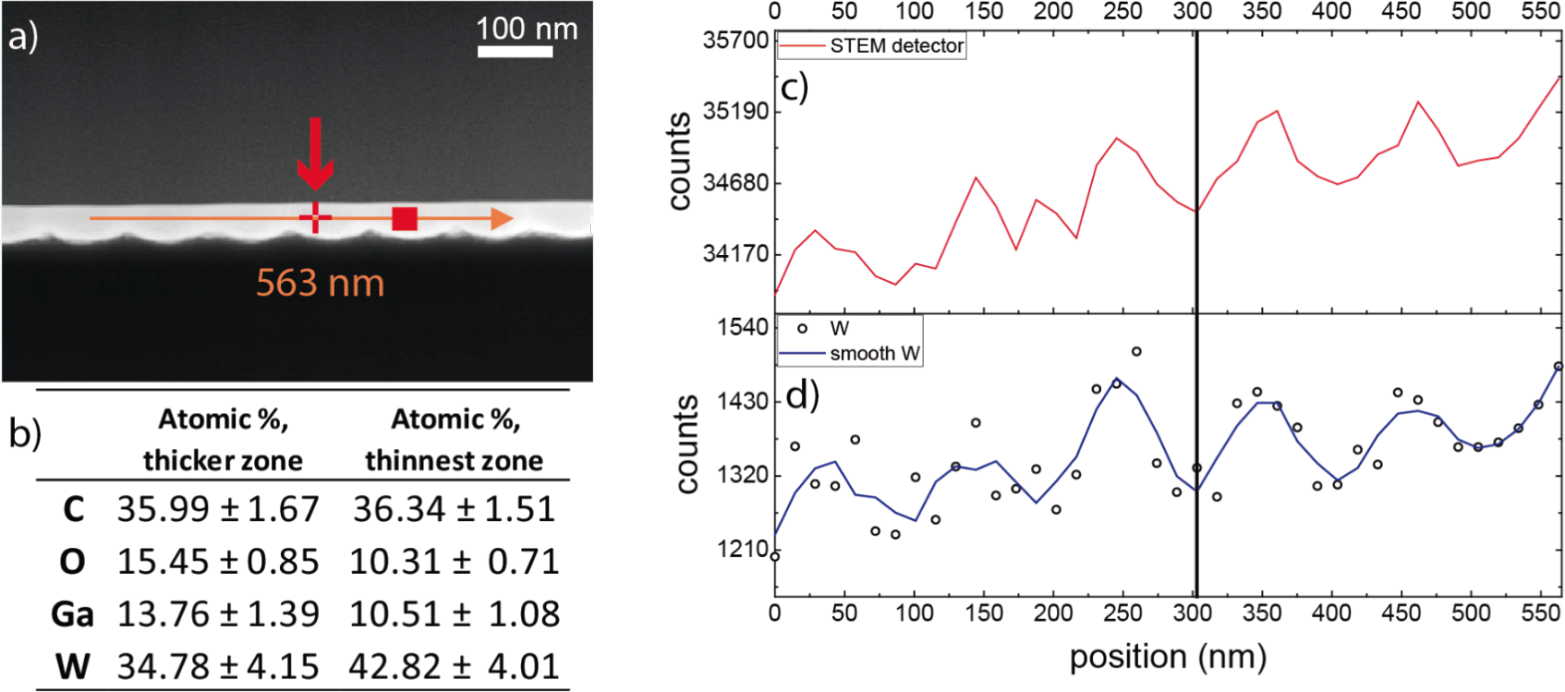
Scanning transmission electron microscopy (STEM) study of the sample with pitch 100 nm. (a) STEM-HAADF image of the sample, including a red arrow signaling the full linear beam scan performed for the compositional analysis shown in (c). (b) Compositional data obtained from EDX spectroscopy measurements performed at the red cross and red square shown in (a) after analyzing all the observed peaks. (c) STEM-HAADF intensity along the red arrow shown in (a). The intensity modulation is related to the slight changes in composition caused by the thickness modulation. The overall slope is a result of the small thickness variation of the lamella where the STEM experiment is carried out. (d) Modulation in the W composition along the red arrow line extracted from the EDX intensity at energy 1774 eV, which corresponds to the W M-edge peak.

The W–C samples were further characterized by means of scanning transmission electron microscopy (STEM) in a 300 kV F30-Tecnai apparatus by FEI. In the STEM experiments performed, the electron beam was scanned inside the nanostructure and parallel to the sample surface, as shown in Figure 3. The total collected high-angle annular-dark-field (HAADF) intensity, which is higher when heavier elements are present, is periodic, indicating a periodic slight variation of the composition. This is expected due to the nature of the growth by FIBID. In the regions directly scanned by the ion beam during growth, a higher amount of Ga ions is expected compared to the regions not directly scanned by the ion beam, where only scattered Ga ions from nearby regions are present. Quantitative analysis of the composition by means of energy dispersive X-ray (EDX) spectroscopy has been performed in the thicker and thinner parts of the deposits. A 3% higher Ga content and 8% lower W content is observed in the thicker parts of the deposits compared to the thinner parts. Such differences give rise to the periodic STEM-HAADF signal. These small differences in composition are not expected to produce nanoscale inhomogeneous superconducting properties. In fact, previous experiments in flat W–C deposits performed by varying the growth beam voltage (from 5 to 30 kV) and the growth beam incidence angle (from 28 to 90°) gave rise to greater changes in the Ga and W content without affecting the measured *T*_c_ [49].

### Magnetotransport experiments

Magnetoresistance measurements in a four-probe configuration as a function of magnetic field (up to 9 T) and temperature (down to 1.9 K) have been carried out with commercial equipment Physical Properties Measurement System (PPMS) from Quantum Design*.* In the following, it is assumed that in our range of measurements the magnetic induction inside the sample, *B*, is equal to μ_0_*H*, with μ_0_ being the vacuum permeability and *H* being the external magnetic field. At the magnetic fields and temperatures under study, the field penetrates practically homogeneously into the superconducting specimen, so that demagnetizing or shielding effects can be neglected.

**Figure 4:**
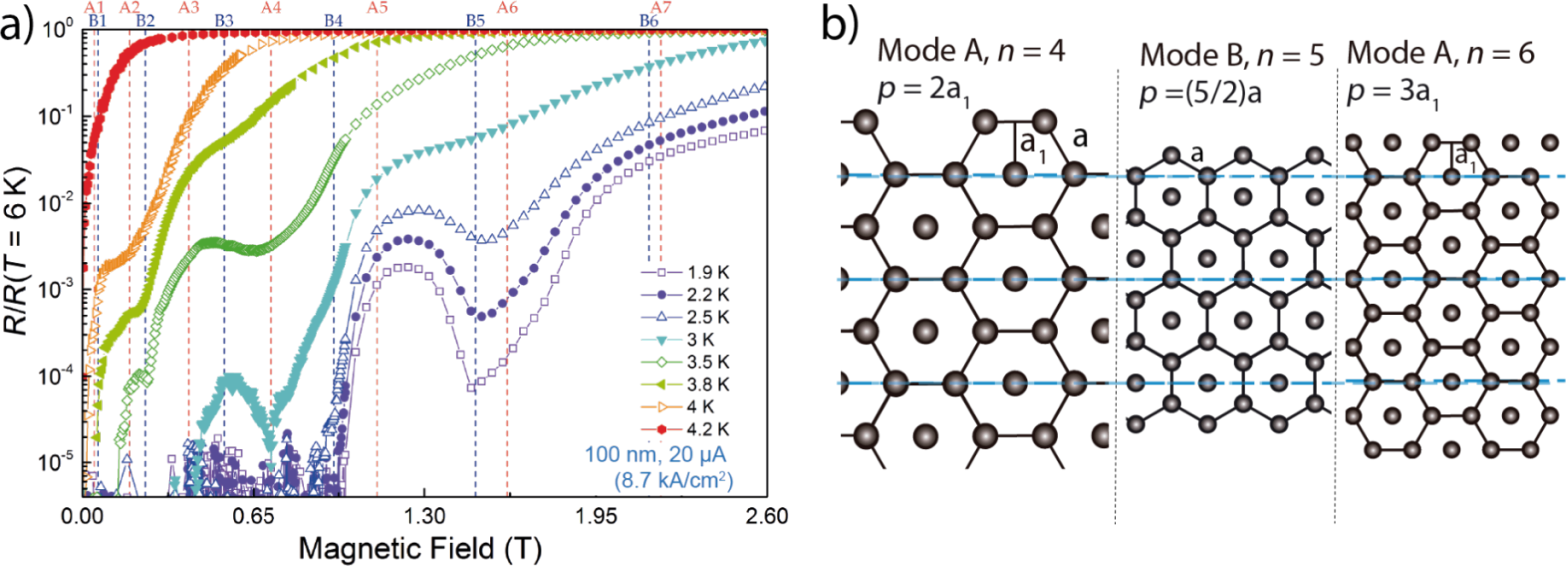
(a) Magnetoresistance curves of the sample with 100 nm pitch at 1.9 K (0.43*T*_c_), 2.2 K (0.49*T*_c_), 2.5 K (0.56*T*_c_), 3 K (0.67*T*_c_), 3.5 K (0.78*T*_c_), 3.8 K (0.85*T*_c_), 4 K (0.9*T*_c_), and 4.2 K (0.94*T*_c_). The measurements were performed with a cureent of 20 μA. Vertical dashed lines in red and blue color indicate, respectively, the theoretical magnetic fields in which Equation 2 (mode A) and Equation 2 (mode B) are satisfied for the sample with 100 nm pitch. (b) Sketch with selected pinning modes.

In Figure 4a, magnetoresistance curves of the sample with 100 nm pitch are displayed. Several resistance local minima are observed, which are interpreted as due to matching effects. A local minimum occurs at 1.56 T, visible in the temperature range between 1.9 and 3 K. As expected, when the minimum is caused by a matching effect, it remains at the same magnetic field over the entire temperature range in which it is visible. For measurements above 3 K, the minimum smears out, indicating that, at that combination of temperature, magnetic field and electrical current, the vortex lattice is too mobile to become pinned at the artificial pinning landscape. Another clear local minimum is visible at 0.67 T between 2.5 and 3 K, which smears out at higher temperature. Other weaker local minima are observed at lower fields in specific temperature ranges.

Previous STM studies on the W–C superconducting films have given evidence for the vortex-lattice arrangement preferably following an Abrikosov triangular lattice [13,15,20–21,50]. Figure 4b shows the two fundamental configurations (modes A and B) for the matching of the vortex lattice to the one-dimensional linear-shape pinning landscape exhibited by the W–C films, which have been theoretically predicted by Martinoli [28]. As we directly imaged by STM in a previous work on a W–C film with a tiny corrugation (less than 1%), the vortex lattice matches to a linear potential following either mode A or B [21]. The relationship between pitch and vortex lattice parameters due to this geometrical pinning can be expressed via the following dependences:

[3]
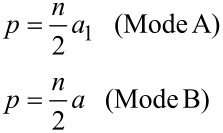


where *p* is the pitch, *a* is the intervortex distance, *a*_1_ is the apothem (*a*_1_ = a√3/2) and *n* = 1, 2, 3… is the order of the matching effect.

Following Equation 1, the intervortex distance depends on *B* in the form B = 
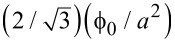
. Consequently, we can calculate the *B* values for which the matching conditions occur, taking into account Equation 1 and Equation 3:

[2]
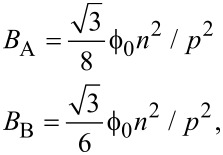


where *B*_A_ and *B*_B_ correspond to the magnetic fields in matching conditions for mode A and mode B, respectively.

In Figure 4a, the vertical lines represent the fields in which the matching conditions from Equation 2 are satisfied for the different matching orders (*n* = 1, 2, 3…) in both matching configurations, A (red) and B (blue). Given that odd orders correspond to pinning lines hosting a different number of vortex in a finite sample (see Figure 4b), the odd matching conditions are expected to be less favorable in samples with width comparable or slightly larger than the intervortex distance.

The local minima in the resistance curves coincide with some of the matching conditions. For example, in the sample with pitch 100 nm, represented in Figure 4a, the local minima at 0.67 T can be assigned to mode A with *n* = 4.

**Figure 5:**
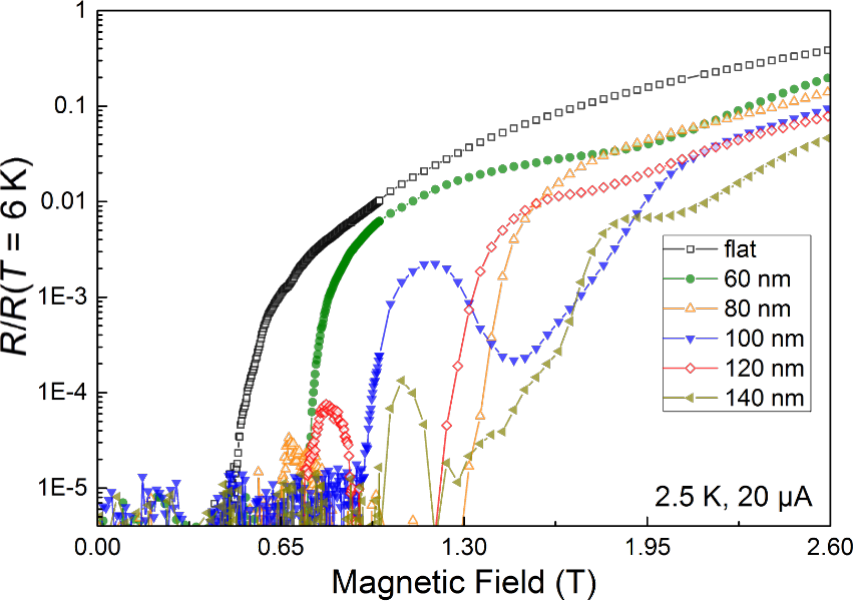
Magnetoresistance curves at 2.5 K of the flat sample, and samples with 60, 80, 100, 120 and 140 nm pitch. The measurements have been carried out using 20 µA, which corresponds to the following current densities: 8.33 kA/cm^2^ (flat), 10.53 kA/cm^2^ (60 nm), 8.70 kA/cm^2^ (80 nm), 8.70 kA/cm^2^ (100 nm), 9.52 kA/cm^2^ (120 nm) and 8.33 kA/cm^2^ (140 nm).

Further support for the explanation of the observed local minima due to vortex matching effects is given by the comparison of the resistance–field curves at 2.5 K for all the investigated samples, as shown in Figure 5. In the flat sample (no corrugation), the resistance is observed to increase monotonously above 0.5 T, ascribed to the dissipation caused by the vortex motion, and without the appearance of resistance local minima. However, the rest of samples, with thickness modulation and associated linear pinning landscape, show resistance local minima at specific magnetic fields that depend on the particular value of the pitch (from 60 to 140 nm) as expected from the matching in Equation 2. Two results should be highlighted. First, in all the samples with thickness modulation, the normalized resistance is smaller than in the flat sample at all magnetic fields. This suggests the relevance of the used pinning landscape to hamper the vortex motion and the associated dissipation. For instance, the sample with 140 nm pitch remarkably shows a normalized resistance at 1.5 T that is three orders of magnitude lower than in the flat sample. Secondly, the resistance local minima are observed up to high magnetic fields. For example, the sample with the maximum pitch, 140 nm, exhibits one local minimum at a field value of 2.2 T, two orders of magnitude larger than in the pioneering studies in the 1970s with micrometric thickness modulation [25,27]. As far as we know, this is a record value regarding resistance–field minima caused by vortex pinning, which is explained by the optimized design of the vortex pinning landscape with the FIB and the intrinsically low, random pinning in these superconductors.

The magnetic fields at which local minima have been experimentally observed in all the thickness-modulated samples are plotted in Supporting Information File 1, Figure S2 as a function of the sample pitch. On the same plot, dashed lines representing the functions corresponding to Equation 2 have been drawn for the different *n* orders. In this way, one can easily link each experimental point to one of the dashed lines in order to assign the probable matching mode and its order. In a minority of cases, the experimental point is close to two nearby curves, making this assignment a bit less safe. We can discuss the particular example of the sample with *p* = 100 nm, shown in Figure 4a, where all the possible modes and *n* values have been annotated in the top part of the figure. The local minimum at 0.67 T is easily assigned to mode A and order *n* = 4 because no other matching option is nearby. In the case of the local minimum at 1.56 T, the matching fields corresponding to mode A and *n* = 6 and mode B and order *n* = 5 are close and the assignment is less safe. It is tempting to ascribe the broad minimum of the resistance at ≈1.5 T to a possible reordering of the vortex lattice or the coexistence of both ordering modes at this magnetic field. Only direct visualization (by STM for example) of the vortex lattice could resolve in this case.

**Figure 6:**
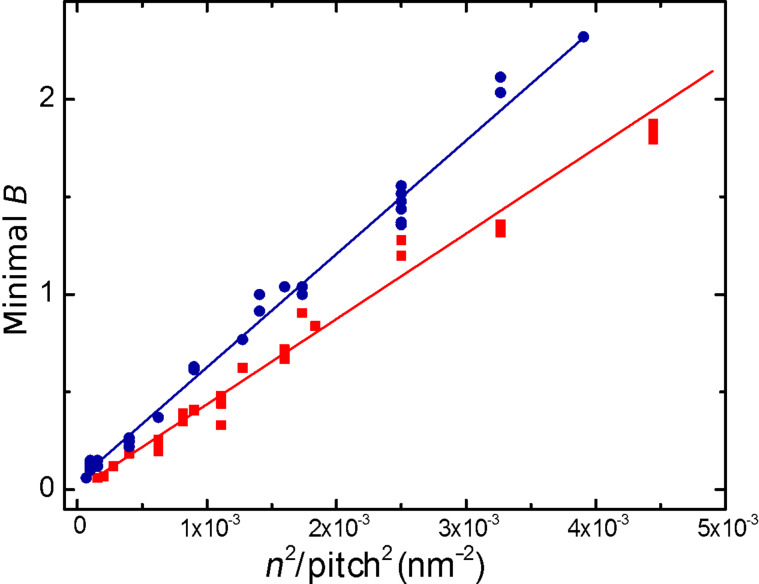
Dependence of the magnetic fields where local minima in the resistance are experimentally observed, as a function of *n*^2^/pitch^2^. The theoretical (linear) dependence expressed by Equation 2 is also displayed for modes A (red) and B (blue).

After carrying out the assignment of all the experimental minima in the magnetic field to a given matching mode and order, a very illustrative mode of representing the data is to draw them as a function of *n*^2^/*p*^2^, together with the theoretical curves (parameter-free) of the dependence of the matching fields, *B*_A_ and *B*_B_, with *n*^2^/*p*^2^, which, according to Equation 2, have a linear relationship with slopes 
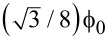
 and 
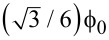
, respectively. The agreement between the theoretical prediction and the experimental result, shown in Figure 6, is good, which reinforces the robustness of the data analysis performed.

**Figure 7:**
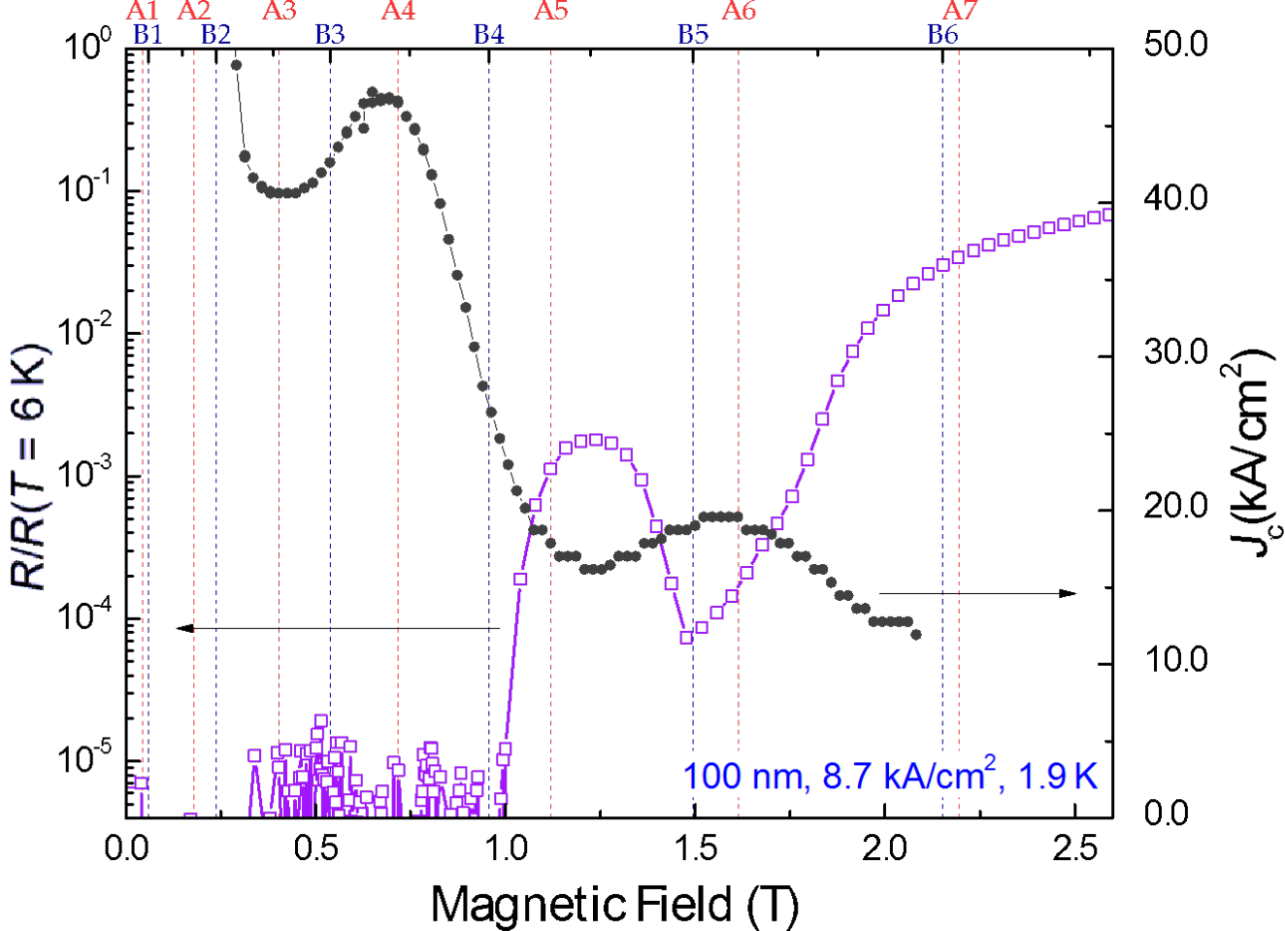
Comparison of the critical current density and the resistance versus magnetic field of the sample with pitch 100 nm at 1.9 K. The matching field corresponding to mode A, *n* = 4 is not observed in the resistance measurement at 1.9 K due to the low value of the resistance but becomes observable at 3 K as displayed in Figure 4a.

Besides, the local resistance minima at matching fields are accompanied by local maxima in the critical current, as shown in Figure 7. The measurement of the critical current as a function of the magnetic field has been carried out for the sample with pitch 100 nm at 1.9 K in order to verify that a minimum in the resistance corresponds to a maximum in the critical current, as previously observed in other superconductors governed by pinning effects [38–39,41,51]. In these measurements, the voltage is limited to a threshold value that corresponds to the crossover from the superconducting to the normal state of the sample. Then, at fixed magnetic field, the current is steadily increased from zero up to the point in which the threshold voltage is reached, which corresponds to the critical current. As can be observed in Figure 7, there is good agreement between the existence of maxima in the critical current and the existence of minima in the resistance–field measurements.

**Figure 8:**
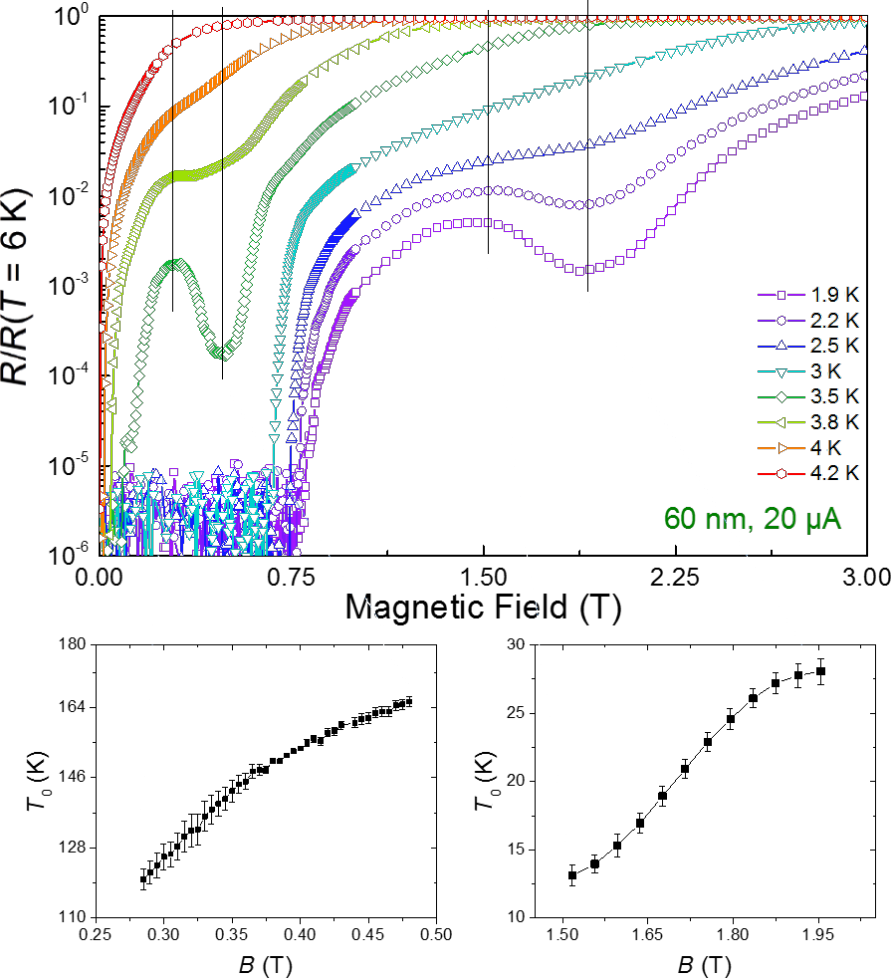
Magnetoresistance measurements for the sample with pitch 60 nm and values of *T*_0_ obtained from the fit to Equation 4 of the descending part of the local minima. The two sets of black lines indicate regions of the fits, given in greater detail below the upper graph.

On the other hand, in between matching fields, the vortex lattice is quite mobile and able to jump over the potential barriers arising from the linear pinning potential. Under matching conditions, the linear pinning potential is able to significantly reduce the vortex motion. The size of the formed potential well can be estimated assuming that around the matching field the resistance follows an activation behavior as a function of temperature:

[4]
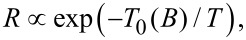


where *T*_0_ is an indicator of the activation barrier [17]. A few selected local minima have been fit to Equation 4, as shown for the sample with 60 nm pitch in Figure 8. For the sample with 60 nm pitch, two local minima have been analyzed, one at matching field around 0.5 T and another one around 2 T. The fit to Equation 4 is realized by analyzing the resistance values at several temperatures under fixed magnetic field. An example of the fits is included in Supporting Information File 1 (Figure S3). The maximum values of *T*_0_ obtained from the fits have been collected in Table 2. One can notice that the energy scale of the pinning potential, expressed through *T*_0_, is six times greater for the 0.5 T matching field than for the 2 T matching field. This can be understood given that the 0.5 T matching field is assigned to the mode A, *n* = 2 whereas the 2 T matching field is assigned to the mode A, *n* = 4. A higher percentage of vortex fall within the pinning lines for mode A, *n* = 2, which can qualitatively explain the difference in the value of *T*_0_.

**Table 2:** Values of *T*_0_ obtained for the fits of the matching fields to Equation 4 in the sample with 60 nm pitch, shown in Figure 8, and in samples with pitch 100 nm and 140 nm.

Pitch (nm)	*B*_min_ (T)	*T*_0_ (K)

60	0.49	166
60	1.88	27
100	0.71	160
100	1.52	37
140	2.11	32

It is remarkable that the pinning effect survives at magnetic fields above 2 T. The observation (or lack) of a matching condition is a result of a subtle balance amongst the vortex lattice stiffness, the Lorentz force, the thermal effects, the intrinsic pinning potential and the artificial pinning potential. Our measurements on the flat sample show that our films have very weak intrinsic pinning, so that the matching effect is largely dominated by the artificial sample nanostructuring. Thus, the combination of the absence of intrinsic pinning and the capability of making very small structures using the FIB is the key to observe matching within such large field and temperature ranges.

## Conclusion

We have found substantial matching effects in W–C superconducting films produced by pinning lines of periodicity between 60 to 140 nm, created by thickness modulation during growth by FIBID. The matching effect between the intervortex distance and the periodicity of the pinning lines gives rise to local minima in the resistance–magnetic field measurements up to high magnetic fields (2.2 T) and over a broad temperature range below *T*_c_. This is a consequence of the low random pinning achieved in this material thanks to the single-step growth with built-in artificial pinning potential. Future STM experiments in similar films will allow the real-space vortex patterns and the changes induced by a current to be viewed. Additional Ginzburg–Landau calculations might help to explain the dissipative behavior close to *T*_c_. Given the broad range of magnetic field and temperature where matching effects have been observed in these W–C films, this material could be interesting to probe dynamical effects of the vortex lattice with one-dimensional pinning potentials. This was recently studied theoretically and showed a rich phase diagram [52]. These W–C films are also convenient for fundamental studies regarding the nature of the vortex-glass to vortex-liquid transition under one-dimensional pinning potential given that previous studies in flat W–C films without artificial pinning have shown good scaling behavior [18]. Additionally, the W–C material could be the building block of arrays of superconducting islands on a normal metal, as was recently investigated for dynamic critical behavior studies [53]. As the pinning properties of these W–C films are highly tunable, it seems feasible to design vortex pinning potentials suitable for Josephson junctions [19] and nano-SQUID devices [54] operating in large field and temperature ranges.

## Experimental

Samples with five different periodicity values of the thickness modulation (60, 80, 100, 120 and 140 nm) and one additional flat sample without modulation were grown. The growth parameters used in the FIBID fabrication process were: *V*_beam_ = 30 kV, *I*_beam_ = 80 pA, beam spot diameter = 39 nm, beam dwell time = 200 ns, *x*-pitch = 100 nm, number of passes = 177988, raster scan mode, precursor temperature = 55 °C, chamber base pressure ≈1 × 10^−6^ mbar, chamber growth pressure ≈1 × 10^−5^ mbar. These parameters have been fixed in all samples whereas the pitch along the *y* direction has been changed between 60 and 140 nm with 20 nm steps. In the manuscript, results are shown for three samples with 60 nm pitch, two samples with 80 nm pitch, three samples with 100 nm pitch, one sample with 120 nm pitch and two samples with 140 nm pitch. The area of the W–C films is 20 × 5 μm^2^ and the distance between the voltage probes is 5 μm. The Pt contacts under the superconducting film have been designed to allow for the growth of the W–C film to be started on a flat surface. For that, first, substrate FIB milling (200 nm deep and 1 μm wide) is performed in order to dig trenches to be subsequently filled with Pt contacts grown by FIBID using a (CH_3_)_3_PtCpCH_3_ precursor. As the surface of the Pt contacts is at the same height as the substrate’s top surface, the superconducting film starts the growth on a flat surface. In the final step, the linear Pt contacts are soldered to the Ti pads by the addition of thick, square, Pt deposits by FIBID.

## Supporting Information

File 1 Current–voltage (*I* vs *V*) behavior.
Assignment of the minima to the matching modes and fits of the resistance–magnetic field curves to thermal-activated behaviour (Equation 4 in the main manuscript).
