# Supplementary material for: Thickness-modulated tungsten–carbon superconducting nanostructures grown by focused ion beam induced deposition for vortex pinning up to high magnetic fields
Source: Beilstein J Nanotechnol. 2016 Nov 14;7:1698–708. doi: 10.3762/bjnano.7.162 (PMC5238659; doi:10.3762/bjnano.7.162)
Supplement: File 1 — Current–voltage (I vs V) behavior. Assignment of the minima to the matching modes and fits of the resistance–magnetic field curves to thermal-activated behaviour (Equation 4 in the main manuscript). [file Beilstein_J_Nanotechnol-07-1698-s001.pdf]

Supporting Information

for

Thickness-modulated tungsten–carbon superconducting nanostructures grown by focused ion beam induced deposition for vortex pinning up to high magnetic fields

Ismael García Serrano[1,2], Javier Sesé[1,2], Isabel Guillamón[3], Hermann Suderow[3], Sebastián Vieira[3], Manuel Ricardo Ibarra[1,2] and José María De Teresa[1,2,4,*]

Address: [1]Laboratorio de Microscopías Avanzadas (LMA), Instituto de Nanociencia de Aragón (INA), Universidad de Zaragoza, 50018 Zaragoza, Spain, [2]Departamento de Física de la Materia Condensada, Universidad de Zaragoza, 50009 Zaragoza, Spain, [3]Laboratorio de Bajas Temperaturas, Instituto de Ciencia de Materiales Nicolás Cabrera, Condensed Matter Physics Center (IFIMAC), Universidad Autónoma de Madrid, 28049 Cantoblanco, Spain, and [4]Instituto de Ciencia de Materiales de Aragón (ICMA), CSIC - Universidad de Zaragoza, 50009 Zaragoza, Spain.

Email: José María De Teresa - deteresa@unizar.es

* Corresponding author

Current–voltage (*I* vs *V*) behavior

In order to determine the critical current as a function of temperature, current-versus-voltage experiments have been carried out in the temperature range between 1.9 K and T_C. As an example, in Figure S1, the results obtained for the sample with pitch=100 nm are shown. T_C is determined from the dependence of the resistance with temperature by using low measuring currents. For all temperatures below T_C, sharp resistance changes are observed at the critical current, as shown in Figure S1(b). The obtained results are in good agreement with previous results by other authors [1], where detailed current-versus-voltage experiments have been reported for W-C nanowires.

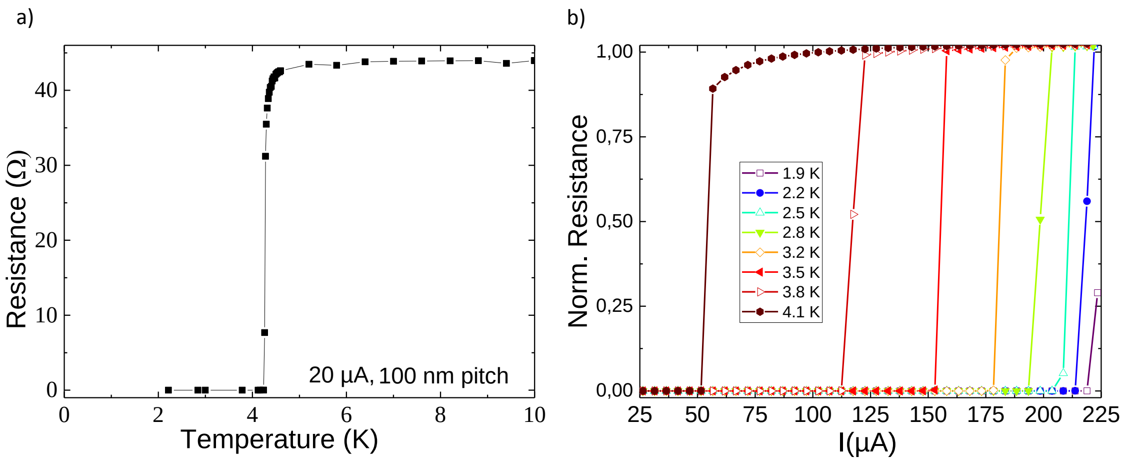

Figure S1: (a) For the sample with 100 nm pitch, resistance-versus-temperature measurements under 20 μA and null magnetic field. T_C is calculated as the temperature for which the resistance in the normal state becomes halved. (b) For the same sample, current-versus-voltage measurements for the determination of the critical current (I_C) in the temperature range 1.9 K to 4.1 K. In the figure, the resistance (normalized to its

value in the normal state) is represented as a function of current. I_C is calculated as the current for which the resistance in the normal state becomes halved.

Assignment of the minima to the matching modes

The magnetic fields at which local minima have been experimentally observed in all the thickness-modulated samples have been plotted in Figure S2 as a function of the sample pitch. On the same plot, dashed lines representing the functions corresponding to relationships (3a) and (3b) have been drawn (in red and blue color respectively) for the different *n* orders. In this way, one can easily link each experimental point to one of the dashed lines in order to assign the probable matching mode and its order.

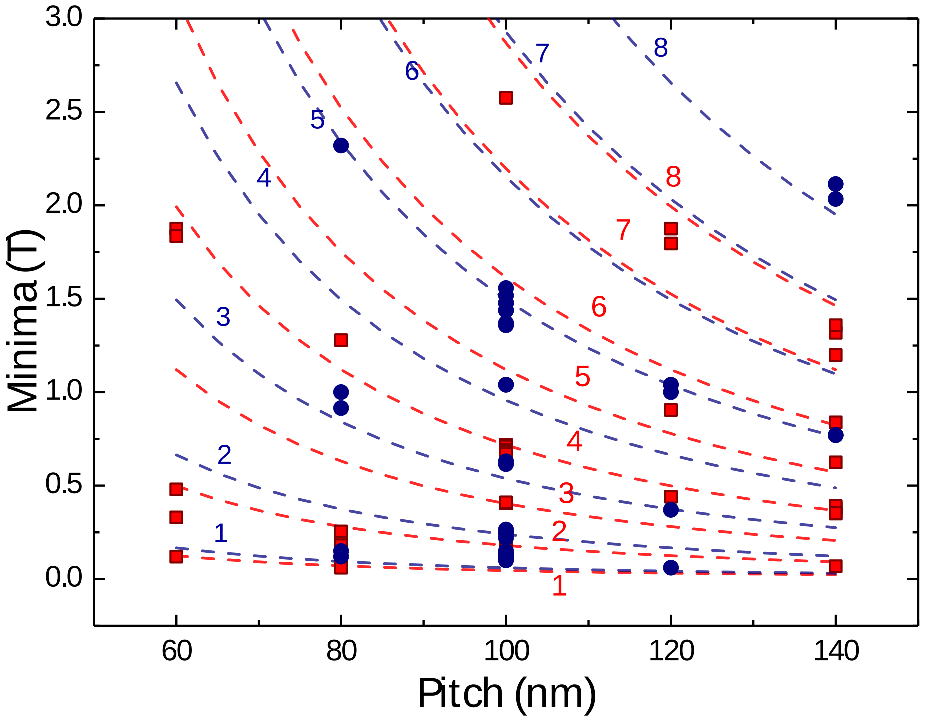

Figure S2: Dependence with the sample pitch of the magnetic fields where local minima in the resistance have been found taking into account the

measurements at all temperatures in all the samples studied. Minima assigned to mode A are represented with (■) symbols and minima considered from mode B with (●) symbols. Dashed lines in red and blue colors represent, respectively, the theoretical dependence predicted by relationships (3a) and (3b) from n = 1 to n = 8.

Fits of the resistance–magnetic-field curves to thermal-activated behavior (Equation 4 in the main manuscript)

The fit to equation (4) has been performed in the following way. First, the magnetoresistance curves which do not display the matching effect are discarded because just a few curves are required for the fit (just two curves are enough). Secondly, the temperature dependence of the resistance values at fixed magnetic field (around the minimum) is fit to equation (4). Typically, this involves three resistance values at fixed magnetic field and one example of this is shown in Figure S3(a), where four fits are shown. The corresponding fields of the fits are indicated by vertical lines in figure S3(b). From the fits, the values of $T_0(B)$ have been determined at each value of B around the minimum. The corresponding T_0-versus-B curves can be drawn, as is shown in Figure 8 in the main manuscript and in the inset of figure S3(b).

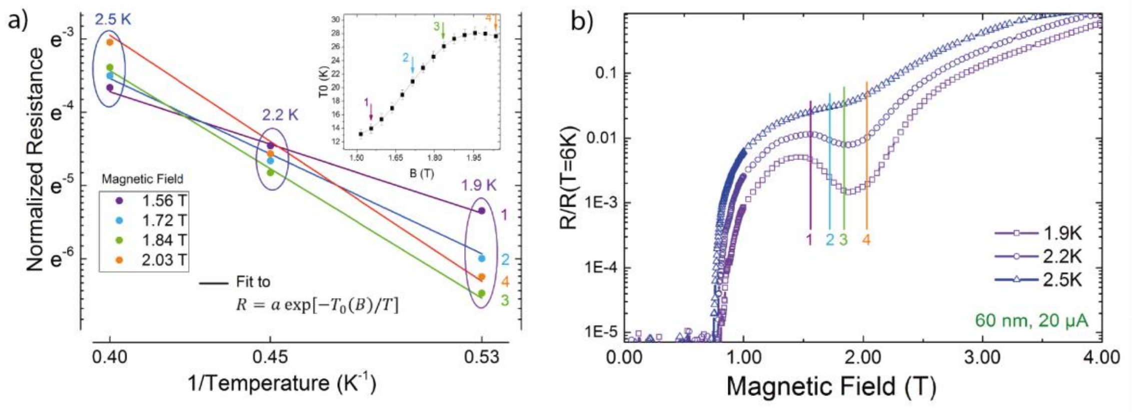

Figure S3: (a) Fits of the resistance-versus-temperature under fixed magnetic field to equation 4 in the main manuscript. The inset shows the values of T_0 obtained from the fits as a function of B**; (b)** Measurements of resistance versus magnetic field at fixed temperature with the four values of B used for the fits shown in (a).

References

1. Sun, Y.; Wang, J.; Zhao, W.; Tian, M.; Singh, M.; Chan, M. H. W. *Sci. Rep.* **2013**, *3*, 2307.
